# Reversible hypoxic‐ischemic encephalopathy post prolonged out‐of‐hospital cardiac arrest: A case series

**DOI:** 10.1002/ccr3.3820

**Published:** 2021-01-22

**Authors:** Fateen Ata, Ammara Bint I Bilal, Omnia Tajelsir Abdalla Osman, Muhammad Awais Arif, Mawahib Elhassan, Tahir hamid, Jassim Al Suwaidi, Hassan Choudry, Galal Abushahba

**Affiliations:** ^1^ Department of Internal Medicine Hamad General Hospital Hamad Medical Corporation Doha Qatar; ^2^ Department of Radiology Hamad General Hospital Hamad Medical Corporation Doha Qatar; ^3^ Department of Cardiology Heart Hospital Hamad Medical Corporation. Doha Qatar; ^4^ Pediatric Gastroenterology Johns Hopkins Medical Institute Baltimore MD USA; ^5^ Department of Cardiology Royal Lancaster Infirmary Hospital Morecambe University Lancaster UK

**Keywords:** anoxic brain injury, cardiopulmonary resuscitation, CPR, hypoxic‐ischemic encephalopathy, neurological recovery, OHCA, out‐of‐hospital cardiac arrest

## Abstract

This article highlights the possibility of positive outcomes associated with prolonged CPR and anoxic brain injury contesting the idea that such patients will invariably end up in a persistent vegetative state.

## 1 INTRODUCTION

Out‐of‐hospital cardiac arrest with prolonged CPR complicated by neurological impairment carries poor prognosis despite appropriate postarrest care. We report three cases with OHCA, prolonged CPR, or a poor neurological status with anoxic/hypoxic brain injury. Hence, complete neurological recovery can be achieved in patients with OHCA irrespective of the CPR length.

Out‐of‐hospital cardiac arrest (OHCA) denotes a loss of the heart's mechanical function and the failure to maintain systemic circulation outside of a hospital. It is challenging to predict OHCA patients' recovery, who subsequently have hypoxic/anoxic brain injury.^1^ These situations produce a prognostic uncertainty with many challenges, such as limiting/withdrawing care or further resuscitations.^2^ Based on the available evidence, it has been observed that prolonged CPR in OHCA patients with hypoxic/anoxic brain injury rarely leads to recovery.^1^ We have reported three cases with OHCA, prolonged CPR, or a poor initial neurological status and confirmed anoxic/hypoxic brain injury in this case series. However, they eventually made an excellent neurological recovery.

## 2 METHODS

We reviewed the cases admitted to the cardiac intensive care unit in the Heart Hospital, Hamad Medical Corporation, Doha, Qatar, retrospectively from 2016 till 2018. A total of 297 cases were admitted as OHCA.

The selection criteria are as follow:

1. Prolonged OHCA of any etiology with ≥ 40 minutes CPR time or a Cerebral Performance Category (CPC) score ≥ 3 at the time of admission.
2. Development of anoxic/hypoxic brain injury after the achievement of ROSC by MRI brain, EEG, or both.
3. Full neurological recovery after a variable period of postresuscitation care (CPC ≤ 1 or Modified Rankin Scale (MRS) ≤ +1).

We included those cases only, which fulfilled all three criteria mentioned above.

## 3 CASE PRESENTATIONS

### 3.1 Case 1

A 68‐years old Egyptian male was admitted to the hospital for an OHCA that developed when he was about to have dinner. The patient was known to have coronary artery disease, paroxysmal atrial fibrillation, type‐2 diabetes mellitus, and hypertension. There were no other symptoms before the collapse. CPR was started by the bystanders and continued later by the EMS (nonphysician‐based ALS certified paramedical staff). Initial ECG revealed ventricular fibrillation (VF). The return of spontaneous circulation (ROSC) was achieved after 44 minutes of CPR. His Cerebral Performance Category (CPC) scale was four, and Modified Rankin Scale was + 5. He was intubated and sedated on‐site by the EMS to protect the airways. Upon arrival at the hospital, he was vitally stable with a BMI of 29.2. The patient was admitted to the cardiac intensive care unit (CICU). ECG on admission revealed sinus rhythm with a 1st degree atrioventricular (AV) block and premature supraventricular complexes. There were no new ischemic changes. Initial 3 sets of troponins were 1063, 760, and 530, 8 hours apart, respectively (normal range 3‐15 ng/L). On the same day (Day 1 of admission), transthoracic echocardiogram (TTE) showed an ejection fraction (EF) of 55%. A coronary angiogram (Day 1) was performed that did not show any significant vascular disease. MRI head (Day 4) demonstrated a slight restriction of diffusion of the cerebral cortex at the parieto‐occipital region likely attributed to hypoxic‐ischemic encephalopathy (HIE). Rhythm disorders, including LQTS and Brugada syndrome, were screened out on ECG. The exact etiology of cardiac arrest could not be identified despite a cardiac MRI was not performed. The patient remained in the CICU for 92 days. Although his clinical course was complicated by acute kidney injury, aspiration pneumonia, and lower GI bleed, his neurological status improved gradually. After resolving ongoing problems, he was transferred to a rehabilitation facility with MRS of + 3 (Day 93) from where he was discharged having a complete neurological recovery with a GCS of 15/15 and MRS score of 0 (Day 141).

### 3.2 Case 2

A 43‐year‐old Syrian male was admitted to the hospital after experiencing OHCA that developed while walking in a park. He was a smoker and did not have any previous disease. CPR was started immediately by his wife and was continued by the EMS (nonphysician‐based ALS certified paramedical staff) for a total duration of 40 minutes [Table 1]. Initial ECG rhythm (Day 1) was pulseless electrical activity PEA, which changed to VF. Post‐ROSC ECG showed ST‐depression in inferolateral leads with ST‐elevation in V1 and AVR. Upon arrival at the hospital, he was afebrile, tachycardic (111 BPM), hypotensive (93/60 mm Hg), and had a BMI of 23.4. Examination revealed an intubated and sedated gentleman with CPC and MRS scales of 4 and + 5, respectively. CT head (Day 1) was unremarkable, and CT pulmonary angiogram (CTPA) (Day 1) did not present any thromboembolic phenomenon. He was admitted to the CICU. The initial clinical impression was NSTEMI vs Brugada syndrome. A TTE (Day 1) showed moderate global hypokinesis of the left ventricle and EF 38%. CT coronary angiogram (Day 1) was unremarkable. MRI head (Day 5) revealed restricted diffusion areas at the high supratentorial cortical regions bilaterally secondary to hypoxic‐ischemic encephalopathy. His neurological condition gradually improved, and he was extubated 19 days later. He was shifted to a general cardiology ward where an ajmaline test (Day 23) was done by the electrophysiology (EP), confirming the diagnosis of Brugada type 1 syndrome, and eventually, implantable cardioverter‐defibrillator (ICD) was placed. Echocardiogram was repeated on day 30 and Ejection Fraction came out normal (>55%). After a total of 47 days, the patient was discharged home with a normal neurological status having GCS and MRS scores of 15/15 and 0, respectively.

**TABLE 1:** Cardiac arrest details of patients

Details	Case 1	Case 2	Case 3
Age	68	43	44
Sex	Male	Male	Female
Cause of arrest	Unknown	Brugada syndrome, type 1	Unknown
ACS as the cause	No	No	No
Locus attached	No	No	No
Total CPR time	44 min	40 min	14 min
CPR by bystander	18 min	NA	10 min
CPR by EMS	26 min	NA	4 min
Number of shocks given	9	9	0
Initial rhythm	VF	PEA	Asystole
Reverted rhythm	Narrow‐complex QRS rhythm	STD: Inferolateral leads, STE: V1 and AVR	Sinus tachycardia with frequent PVC
CAG	Unremarkable	Unremarkable	Unremarkable
MRI head	HIE	HIE	HIE
EEG	NA	NA	Low voltage
CPC scale at admission	4	4	4
MRS at admission	+5	+5	+5
GCS at discharge	15/15	15/15	15/15
MRS at discharge	0	0	0
Total hospital days	141	47	80

Abbreviations: ACS, acute coronary syndrome; CAG, coronary angiogram; CPR, cardiopulmonary resuscitation; EEG, electroencephalogram; EMS, emergency medical services; HIE, hypoxic‐ischemic encephalopathy; NA, not available; NSTEMI, non‐ST‐elevation myocardial infarction; PEA, pulseless electrical activity; PVC, premature ventricular complexes; STD, ST‐depression; STE, ST‐elevation; VF, ventricular fibrillation.

### 3.3 Case 3

A 44‐year‐old Romanian female was admitted to the hospital after experiencing an OHCA that developed when she was at the airport. She had a history of arrhythmias, but the exact diagnosis was unknown. She was advised for an ICD insertion in her home country, as reported by her husband. The patient was resuscitated by the bystanders and later by the EMS (nonphysician‐based ALS certified paramedical staff) with a CPR of 14 minutes [Table 1]. Initial ECG showed asystole. The EMS intubated her. When brought to the hospital, she had CPC and MRS scores of 4 and + 5, respectively. She was afebrile, tachycardic (105 BPM), and hypotensive (97/65 mm Hg) with a BMI of 21.7. Her Pupils were dilated but reactive. The neurological examination revealed spontaneous eye‐opening, no response to painful stimuli, but the reflexes were preserved. The ECG (Day 1) after ROSC showed sinus tachycardia with frequent premature ventricular complexes (PVC). She was admitted to the CICU. CT head (Day 1) was unremarkable for any acute intracranial hemorrhage, and a CTPA (Day 1) did not manifest pulmonary embolism. A TTE (Day 1) revealed global wall hypokinesia and EF of 37%. MRI head (Day 4) unveiled bilateral parieto‐occipital cortical and subcortical HIE. An electroencephalogram (EEG) (Day 5) demonstrated low voltage, with no status epilepticus or epileptic discharges. Eight days after admission, her mental status started improving with spontaneous hand movements. She received multiple sessions of cardiac rehabilitation, physical therapy, and occupational therapy. She was extubated and shifted to the general cardiology ward after 12 days with a GCS of 10/15 and MRS + 4. During the patient's stay in the CCU and the general cardiology ward, she developed short runs of nonsustained ventricular tachycardia. There was no evidence of significant coronary artery disease, and the calcium score was zero on CT coronary angiogram (Day 21). The exact etiology of her cardiac arrest could not be established as the patient, and her family refused any further evaluation by the EP team, including EP study. After 80 days, the patient gradually had a complete neurological recovery and was discharged with a GCS of 15/15 and MRS 0.

## 4 RESULTS

The three reported cases had a median CPR time of 40 minutes with varying initial rhythms (VF, PEA, and asystole) and different underlying etiologies of the cardiac arrest. All of them had neurological impairment diagnosed clinically by the neurology teams' assessment and confirmed either brain MRI, EEG, or both [Table 1]. All the data from the arrest time until the patients' discharge was analyzed, including the EMS details upon arrival, the initial rhythm, whether the CPR was manual or by LUCAS Lund University Cardiopulmonary Assist System (LUCAS), laboratory, and imaging parameters. After a median recovery time of 80 days, all the cases eventually made an excellent neurological recovery with normal functional status.

## 5 DISCUSSION

Cardiac arrest is among the leading causes of mortality worldwide, making it a significant health burden, with above 0.5 million deaths per year in North America alone.^3^ Overall, the average survival rate is around 7‐8 percent.^1^ Although there have been advances in CPR protocols over the years, the mortality rate in OHCA still approaches 75 percent. In those who survive, the morbidity burden, especially neurological injury, is high.^4^, ^5^ Various factors contribute to the mortality and extent of morbidity in patients with OHCA. Duration of CPR is considered extremely consequential in determining mortality and long‐term morbidity, including the extent of neurological injury in such patients.^4^

Hallstrom et al analyzed compounding comorbid conditions (including myocardial infarction, heart failure, diabetes, hypertension, and cancers) and their effects on patients' survival rates with cardiac arrest.^6^ In addition to mortality, these factors contribute substantially to morbidity, particularly neurological damage. Neurological impairment, especially when severe, has been described as morbidity worse than mortality.^7^ Hence, all efforts should be made to improve neurological function as much and whenever possible.

Various existing modalities are used to assess and confirm the quantity and quality of neurological damage in patients with cardiac arrest such as Electroencephalogram (EEG), biomarkers such as Neuron‐specific enolase (NSE), Near‐infrared spectroscopy (NIRS), Computed tomography (CT) scan, and Magnetic Resonance Imaging (MRI) of the brain.^8^ Presently, MRI provides a maximum benefit if performed 2‐5 days after the neurological insult (which is secondary to OHCA in our cases), based on previous studies.^9^

A few predictors of functional neurological recovery that have been studied in the past include the characteristics of EEG in the first 12 hours after ROSC, features of reactivity on EEG in the first few h, and absence of diffusion‐weighted imaging changes on MRI within the first‐wk post‐ROSC.^9^, ^10^ However, this aspect of post‐OHCA care has not been studied as extensively as required to formulate guidelines. Following the uncertainty surrounding neurological recovery post prolonged CPR (more than 15 minutes), the status quo accepts unfavorable outcomes and generally poor prognosis with a six‐wk survival approaching almost zero percent.^11^

In the event of OHCA, one of the significant factors in determining survival is the initiation of CPR by bystanders or trained first responders before the arrival of EMS. However, these circumstantial events and their effects have not been thoroughly studied to formulate valid conclusions.^12^ Usually, bystander CPR (if done by untrained personnel) is not as effective as experienced healthcare personnel.^13^ In our cases, the patients had at least 10 minutes of bystander CPR before the arrival of EMS. This indicates that various other factors besides the quality or duration of CPR determine patients' overall outcome with OHCA. Hence, it is necessary to reanalyze the mortality and morbidity effects of bystander CPR and CPR duration in patients who resultantly have hypoxic brain insult.

In patients with OHCA who end up in a vegetative state following brain hypoxia, the level and extent of supportive care to offer and the decisions related to end‐of‐life care remain vital challenges faced by the treating physicians. Family is usually involved in resuscitation decisions once a vegetative state is deemed irreversible, and there is uncertainty regarding the prognosis (considering a prolonged cardiac arrest and unchanging neurological condition while in hospital).^2^ Proper identification of prognosticators of long‐term mortality and morbidity becomes very pivotal to reach an appropriate decision and reduce the burden on healthcare systems.

Based on this case series, we suggest that patients who develop hypoxic/anoxic brain injury after prolonged CPR still have chances of a better prognosis than currently believed. Some of these patients may regain complete neurological function, and while the duration of CPR is a considerable contributor, it is not the only factor coming into play regarding prognostic outcomes. Further prospective studies are required to assess neurological damage's reversibility after prolonged CPR in patients with OHCA. Significant covariates (especially cause of arrest, patient's age and comorbidities, days of hospital and critical care stay, quality of CPR provided, use of LUCAS machine, and other unknown covariates) need to be adjusted, and their effects analyzed to understand the mechanism of complete neurological recovery in these patients.

Prospective studies can also provide valuable information required to make decisions for end‐of‐life care in such patients, including but not limited to the number of hospital days without a neurological improvement, the rate and degree of neurological recovery, and prevalence of achieving complete neurological function before limiting or discontinuing the supportive care and resuscitative efforts in the face of another cardiac arrest.

## 6 CONCLUSION

Out‐of‐hospital cardiac arrest with prolonged CPR and evidence of anoxic/hypoxic brain injury may still result in complete neurological recovery; some cases might require protracted in‐patient care to regain full neurological function. Further studies are needed to elaborate more on the factors associated with complete neurological recovery. With this case series, we have highlighted positive outcomes associated with prolonged CPR and anoxic brain injury contesting the idea that such patients will invariably end up in a persistent vegetative state.

## CONFLICT OF INTEREST

This manuscript is original work and has not been submitted or is not under consideration for publication elsewhere. All the authors have reviewed the manuscript and approved it before submission. None of the authors have any conflict of interest from publishing this work.

## AUTHOR CONTRIBUTIONS

FA: manuscript writing, literature review, review, and approval of the final manuscript. OO: case writing, literature review, review, and approval of the final manuscript. ME: case writing, literature review, critical review of the manuscript with revisions, review, and approval of the final manuscript. AB: case writing, literature review, review, and approval of the final manuscript. MA: manuscript writing, literature review, review, and approval of the final manuscript. TH: literature review, critical review of the manuscript with revisions, review, and approval of the final manuscript. GA: determined eligibility and case identification, manuscript writing, literature review, critical review of manuscript and revisions, and approval of the final manuscript. HC: literature review and revisions in the manuscript. JS: literature review and revisions in the manuscript.

## ETHICAL APPROVAL

This work was approved by the Medical Research Center (MRC) Qatar before submission.
